# Comparison of Numerical Simulation Techniques of Ballistic Ceramics under Projectile Impact Conditions

**DOI:** 10.3390/ma15010018

**Published:** 2021-12-21

**Authors:** Pawel Zochowski, Marcin Bajkowski, Roman Grygoruk, Mariusz Magier, Wojciech Burian, Dariusz Pyka, Miroslaw Bocian, Krzysztof Jamroziak

**Affiliations:** 1 Military Institute of Armament Technology, Prym. S. Wyszynskiego 7, 05-220 Zielonka, Poland; zochowskip@witu.mil.pl; 2 Institute of Mechanics and Printing, Faculty of Mechanical and Industrial Engineering, Warsaw University of Technology, Narbutta 85, 02-524 Warsaw, Poland; marcin.bajkowski@pw.edu.pl (M.B.); roman.grygoruk@pw.edu.pl (R.G.); 3 Łukasiewicz Research Network—Institute of Non-Ferrous Metals, Sowinskiego 5, 44-100 Gliwice, Poland; wojciech.burian@imn.gliwice.pl; 4 Department of Mechanics, Materials Science and Engineering, Faculty of Mechanical Engineering, Wroclaw University of Science and Technology, Smoluchowskiego 25, 50-370 Wroclaw, Poland; dariusz.pyka@pwr.edu.pl (D.P.); miroslaw.bocian@pwr.edu.pl (M.B.)

**Keywords:** ballistic impact, ceramic armor, numerical simulation, finite element method, smoothed particles hydrodynamics

## Abstract

This article presents an analysis of the effectiveness of available numerical techniques in mapping the characteristic behavior of ballistic ceramics under projectile impact conditions. As part of the work, the ballistic tests were performed on the layered ceramic/steel composite armor and tested with the 7.62 × 39 mm, armor-piercing incendiary (API) BZ projectile. The experimental tests were then mapped using computer simulations. In numerical analyses, four different techniques were used to describe cubic ceramic tiles Al_2_O_3_ placed on the ARMOX 500T steel backing plate, i.e.,: the Finite Element Method without Erosion (FEM), Finite Element with erosion (FEM + Erosion), Smoothed Particles Hydrodynamics (SPH) and a hybrid method that converts finite elements to SPH particles after exceeding the defined failure criteria (FEM to SPH conversion). The effectiveness of the individual methods was compared in terms of quality (mapping of characteristic phenomena occurring during the penetration process), quantity (bulge height of the backing plate) and time needed to complete the calculations. On the basis of the results of the experiments and numerical simulations, it was noticed that the most accurate reproduction of the phenomenon of ballistic impact of AP projectiles on ceramic/steel composite armor can be obtained by using a hybrid method, incorporating the conversion of finite elements into SPH particles. This method should be used in cases where accuracy of the results is more important than the time required to complete the calculations. In other situations where the purpose of the calculation is not to determine, for example, the exact value of penetration depth but only to observe a certain trend, the FEM method with defined erosion criteria (variant 2), which is more than 10 times faster, can be successfully used.

## 1. Introduction

Continuous development of kinetic energy projectiles causes an increase in the demands on modern ballistic protection equipment. The effectiveness of armor is defined as the ability to absorb and dissipate the impact energy of a specific type of projectile [1]. In addition, trends in the modern armament industry impose requirements that the increase in the protective effectiveness of the armor should not increase its mass. Therefore, the search for optimal solutions is directed towards composite and multilayered structures. Layers in such constructions can be made of various materials [2,3,4] that are selected and arranged in a proper way to prevent perforation of the armor [5,6].

One of the basic materials used in layered armors is ballistic ceramic [7,8,9] exhibiting significantly higher hardness and wave impedance than steel [10,11]. Ceramic elements in armor structures are mainly used as a frontal layer. Therefore, their task is to crush or blunt the impacting projectile and to absorb a part of its kinetic energy, mainly through the mechanism of brittle cracking. The ceramic layer in the armor is usually composed of tiles with the base of a quadrilateral or hexagon [12,13], or in the form of spurs and elements resembling the natural structure of crustaceans, and carbide armor [14,15,16,17]. Since ceramic elements are significantly damaged during projectile impact, they are usually small in size in order to reduce the risk of hitting the damaged area again and thus the loss of the protective capacity of the armor, especially during multi-hit firing.

Analyses of the protective effectiveness of layered composite armors containing ceramic tiles are focused on analytical [18], combined analytical-experimental [19,20], and numerical methods that, recently, have become very popular [21,22,23]. Computational techniques analyze complex phenomena based on the basic principles of mechanics. Numerical methods allow researchers to shorten the time and reduce costs of testing new materials and constructions, e.g., by selecting the potentially best variants and limiting the number of physical models subjected to experimental research. Simulations allow researchers to collect more information about the entire analyzed process, e.g., pressure, density, or velocity distribution at different times.

In the literature there are many examples of using of numerical methods for the analysis of highly dynamic phenomena. Computer programs such as LS-DYNA, ABAQUS, ANSYS AUTODYN, etc. [24,25] are commonly used to assess the ballistic effectiveness of ceramic and composite armor. In [26], in the example of 7.62 × 54 R mm B32 armor-piercing incendiary (API) projectile impact, the process of impactor energy dissipation was analyzed using the finite elements method (FEM) implemented in the LS-DYNA code. In [27], the phenomenon of 7.62 × 51 mm armor-piercing (AP) (M61) projectile-firing was described using a hybrid method combining Lagrange and Euler formulations—Arbitrary Lagrangian Eulerian (ALE). In [28], the impact of a 7.62 × 39 mm FMJ M43 projectile on composite armor reinforced with α-AL_2_O_3_ particles was realized using the approach based on combining FEM and SPH methods implemented in ABAQUS software.

As can be seen, the accurate numerical representation of all mechanisms occurring during the process of penetration of ceramic tiles by an armor-piercing projectile is still challenging due to brittle cracking of the layers and the high degree of damage to them under ballistic impact conditions. Therefore, as has been shown in the literature, hybrid methods of modelling have recently been dominant in analyses of those processes [23,29,30]. In such approaches, several different methods and element formulations (e.g., Lagrange, Euler, ALE, SPH, etc.) are combined within a single analysis. Obviously, each of these methods exhibits its own specific advantages and disadvantages. In Lagrange’s description, the spatial mesh of the body moves and deforms with it. The disadvantage of the method is the slowing down or interrupting of calculations in case of the simulation of phenomena with large body deformations (a problem of degenerated mesh elements), which forces the user to use the algorithm to remove deformed elements (erosion). Application of the erosion algorithm prevents the mass and energy of the system from being preserved. In the simulation of a ballistic impact phenomenon, this may lead to significant differences in results between the simulation and the experiment. In order to avoid problems related to the removal of a large number of finite elements, the FEM in Euler’s description is a helpful method. The mesh in this method is fixed against the background of the moving material of the simulated body. This method is preferred for simulating fluids and materials with high plasticity. It is not recommended for the mapping of brittle materials, which are destroyed by cracking.

The disadvantages of the above-mentioned methods may be compensated for by the use of mesh-free methods, e.g., the particle-based SPH method [31]. Each of the above-mentioned methods can be used alternately in order to obtain the results closest to the experimental findings. The possibilities of using approaches combining several methods have been described in [32,33,34], where the phenomenon of a collision between a bird and an airplane has been analyzed.

In this article, an attempt was made to select an optimal technique of numerical reproduction of the characteristic behavior of ballistic ceramics under projectile impact conditions. It should be noted that the vast majority of previously published works in this field refer to the use of one method or the other, but the accuracy and effectiveness of individual methods were not compared. Additionally, the novelty of the presented study lies in the fact that particular attention was paid to reproducing the phenomena of generation and propagation of cracks in ceramics as accurately as possible. These mechanisms have a decisive influence on the high efficiency of the ceramic layers in armor applications. Therefore, their correct numerical reproduction is crucial for the definition of an accurate numerical model of ballistic impact phenomena on ceramic layers. For this purpose, ballistic tests were carried out on the layered ceramic/steel composite armor impacted by the 7.62 × 39 mm API(BZ) projectile. Then, the experimental tests were mapped using computer simulations. In numerical analyses, four different techniques were used to describe cubic Al_2_O_3_ ceramic tiles, placed on an ARMOX 500T steel backing plate. FEM without erosion, FEM with erosion, the SPH method and a hybrid method, using the conversion of finite elements to SPH particles after reaching defined failure criteria, were used. The effectiveness of individual methods was compared both in terms of the quality of mapping of characteristic phenomena occurring during the penetration process and the time necessary to complete the calculations.

## 2. Object and Methodology of Tests

### 2.1. Test Materials

**Figure 1:**
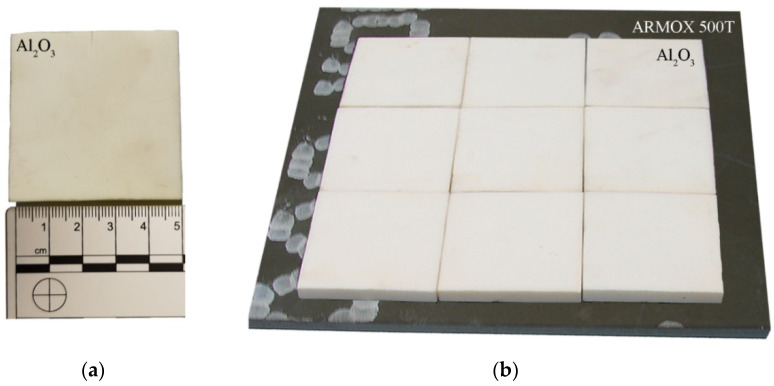
Samples for testing: (**a**) ceramic tile; (**b**) armored plate ARMOX 500T.

The layered ceramic/steel composite armor was analyzed in the article. Al_2_O_3_ alumina ceramic tiles with dimensions of 50 mm × 50 mm and a thickness of 5 mm were used in the study. The tiles were placed on a 4 mm-thick ARMOX 500T steel backing plate (see Figure 1). The properties of Al_2_O_3_ ceramics used in these experiments are presented in Table 1, compared to other types of special ceramics used in armor and to rolled homogeneous armor steel (RHA).

**Figure 2:**
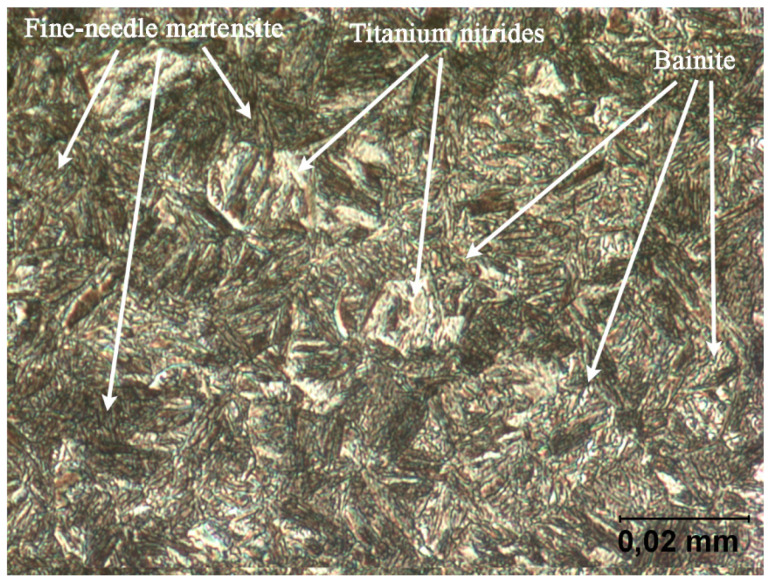
Structure of the armor plate A500T–tempering martensite. Light microscopy, etched with 5% HNO_3_.

The parameters of SSAB’s ARMOX 500T armor steel, one of the most popular steel grades of this type, were summarized in Table 2. The material has a martensitic structure (see Figure 2), and exhibits high hardness as well as a balanced combination of strength and ductility parameters.

On the basis of microscopic observation, it was concluded that the ARMOX 500T structure consists of fine needle tempered martensite with a small amount of bainite (see Figure 2). Moreover, local precipitation of titanium nitrides was observed in the structure, the content of which is not included in the manufacturer specification.

### 2.2. Ballistic Test

**Figure 3:**
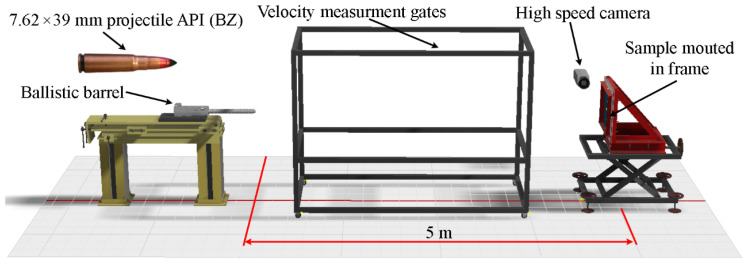
Diagram of the ballistic test stand for the tests of ceramic tiles.

Experimental tests were carried out in the ballistic tunnel with the use of a ballistic barrel cal. 7.62 × 39 mm, with a length of 650 mm. The elements included in the test stand were shown in Figure 3. During the ballistic tests, the distance between the barrel outlet and the backing plate was 5 m. The axis of the barrel formed an angle of α = 90° in relation to the surface of the backing plate (see Figure 3). The 4 mm-thick backing plate was mounted on a bracket and its edges were fixed by the pressure of the bolted frame. The 5 mm-thick ceramic tiles were glued to the surface of the backing plate (see Figure 3). The backing plate was also a “witness” plate, i.e., on the basis of the value of its deformation (bulge height), the protective effectiveness of the tested ceramic tile was assessed. Each of the shots was recorded with a high-speed camera. The velocity of the projectile was measured by means of measuring gates.

**Figure 4:**
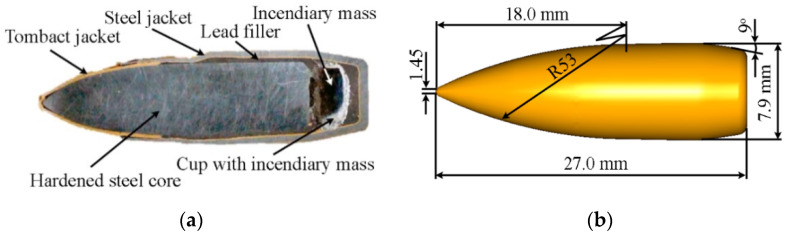
This 7.62 × 39 mm API(BZ) projectile: (**a**) cross-section; (**b**) projectile geometry.

During the tests, the samples were impacted by the 7.62 × 39 mm API(BZ) projectile belonging to the second level according to STANAG 4569 [38]. This projectile is usually used against lightly armored targets. It consists of a tombac-plated steel jacket, a tombac jacket, lead filler, a hardened steel core and an incendiary cup placed behind the core (see Figure 4).

The initial velocity of the projectile is 695 m/s ± 20 and the mass is 7.8 g, which corresponds to an impact energy of about *E_i_* ≅ 1.9 kJ.

### 2.3. Methodology of Numerical Simulation

**Figure 5:**
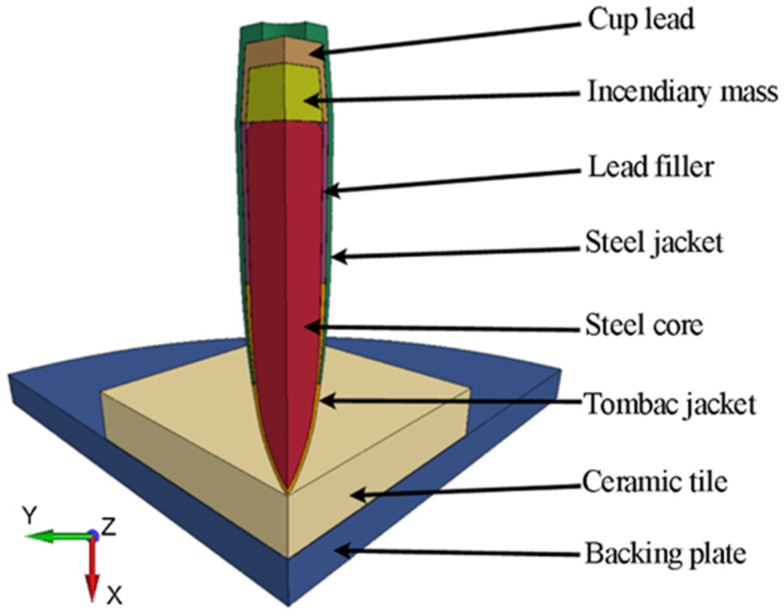
Geometrical model and components of the armor and projectile system.

Numerical simulations of the ballistic impact of a 7.62 × 39 mm API projectile (BZ) on the ceramic/steel composite armor were performed on LS-Dyna software. Individual components of the simulation were arranged in a way corresponding to the experimental research (Figure 5). Al_2_O_3_ ceramic tiles with dimensions of 50 × 50 × 5 mm were placed on the ARMOX 500T steel backing plate with dimensions of 500 × 500 × 4 mm.

Four variants of the simulation were carried out, varied with the method ceramic tile modeling:

(1) Variant 1—ceramic tile described by means of finite elements without erosion;
(2) Variant 2—ceramic tile described by means of finite elements with erosion;
(3) Variant 3—ceramic tile described by means of SPH particles without erosion;
(4) Variant 4—ceramic tile described by means of hybrid elements—finite elements converted to SPH particles after exceeding the failure criteria.

A constitutive model based on the Johnson–Cook (J-C) equation was used to describe metallic components of the simulation [39,40]. This model reproduces the behavior of metals under high strain rates in both elastic and plastic ranges, including material strengthening and thermal softening. The relatively easy procedure of determining constants makes the Johnson–Cook model one of the most frequently used for metals. Thanks to this, values of parameters of the J–C equation are available for a large number of different metals. The yield strength in the J–C model is a function of effective plastic strain, strain rate and temperature:

(1) $$\sigma_{y}=\left(A+B{\bar{\varepsilon }}^{p^{n}}\right)\left(1+Cln{\dot{\varepsilon }}^{*}\right)\left(1-T^{*}{}^{m}\right),$$

(2) $${\dot{\varepsilon }}^{*}=\frac{{\dot{\bar{\varepsilon }}}^{p}}{{\dot{\varepsilon }}_{0}},$$

(3) $$T^{*}=\frac{T-T_{room}}{T_{melt}-T_{room}},$$

where: *A* = yield strength; *B* = strengthening constant; *C* = strain rate constant; *n* = strengthening exponent; *m* = thermal softening factor; ${\bar{\varepsilon }}^{p}$ = effective plastic strain; ${\dot{\bar{\varepsilon }}}^{p}$ = strain rate; ${\dot{\varepsilon }}^{*}$ = dimensionless effective strain rate; ${\dot{\varepsilon }}_{0}$ = reference value for the strain rate; $T^{*}$ = homologated temperature (dimensionless); *T_room_* = room temperature; *T_melt_* = melting point; *T* = current temperature.

In this work, two types of J–C strength model were used: a simplified model (*MAT_098) and a modified model (*MAT_107). The modified model has been supplemented with the J–C failure model, in which the strain occurring at the moment of failure is described by the following relation:

(4) $$\varepsilon_{f}=\left[D_{1}+D_{2}e^{D_{3}\sigma^{*}}\right]\left[1+D_{4}ln{\dot{\varepsilon }}^{*}\right]\left[1+D_{5}T^{*}\right],$$

(5) $$\sigma^{*}=\frac{p}{\sigma_{eff}},$$

where: *D*_1_, *D*_2_, *D*_3_, *D*_4_ and *D*_5_ = failure parameters; σ* = stress triaxiality factor; *p* = pressure, *σ_eff_* = effective stress.

The stress state in the material, defined by the parameters *D*_1_, *D*_2_ and *D*_3_ in the J–C failure model, has a decisive influence on the value of the failure strain. The J–C failure model is “instantaneous”—after the failure of an element (part) is detected, its stiffness and strength are reduced to zero. The failure occurs when the failure parameter reaches a value of unity:

(6) $$D=\sum^{\;}\frac{\Delta {\bar{\varepsilon }}^{p}}{\varepsilon_{f}}=1,$$

where: $\Delta {\bar{\varepsilon }}^{p}$ = increase in the value of effective plastic strain; *ε_f_* = failure strain.

The Johnson–Holmquist model (*MAT_110) was used to describe the ceramic materials. The Johnson–Holmquist relationship defines a brittle material model consisting of a polynomial state equation. A special feature of this model is that it uses two strength limits of the material: for undamaged material and for damaged material. Both values are determined by pressure *p* and strain rate $\dot{\varepsilon }$.

The equations of the strength model J–H are as follows:

- for undamaged material:

(7) $$\sigma_{i}\left(p,\dot{\varepsilon }\right)=A_{J-H}\sigma_{HEL}{\left(\frac{T-p}{p_{HEL}}\right)}^{D_{2}}\left(1+C_{J-H}ln\left(\frac{\dot{\varepsilon }}{{\dot{\varepsilon }}_{0}}\right)\right),$$

- for damaged material:

(8) $$\sigma_{i}\left(p,\dot{\varepsilon }\right)=B_{J-H}\sigma_{HEL}{\left(\frac{p}{p_{HEL}}\right)}^{M_{J-H}}\left(1+C_{J-H}ln\left(\frac{\dot{\varepsilon }}{{\dot{\varepsilon }}_{0}}\right)\right),$$

- equation of the failure model:

(9) $$\varepsilon_{p}^{f}\left(p\right)=D_{1}{\left(\frac{T-p}{p_{HEL}}\right)}^{D_{2}},$$

where: *p_HEL_* = pressure for the yield point of Hugoniot; *σ_HEL_* = stress for the actual yield point of Hugoniot; *A_J-H_* = strength constant of the intact material; *N_J-H_* = strength exponent of the intact material; *C_J-H_* = strain rate constant; *B_J-H_* = strength constant of the damaged material; *M_J-H_* = strength constant of the damaged material; *D*_1_ and *D*_2_ = the constant and the exponent of failure respectively.

The data for the numerical models were taken from the literature and from our own library of materials developed on the basis of our own research characterizing the materials [41,42,43,44,45,46]. The list of applied parameter values is presented in Table 3 and Table 4.

In addition, for individual material models, appropriate erosion criteria (*MAT_000-ADD_EROSION) have been applied, based on the stress and strain limits that can occur in the materials. When the stress/strain limit value was reached, the element was removed from the calculation. In this way, the problem of degenerated elements, which slows down and, in extreme cases, even makes it impossible to continue with the calculation, was eliminated. Since ceramic tiles show very low tensile strength, their erosion criterion based on the limiting volumetric strain was determined as 5% (VOLEPS = 0.05) similarly to [47,48], in which the authors adopted the limiting value of strain as 6%.

For the components of the analyzed phenomenon of API projectile impact on ceramic/steel composite armor, the size of the finite elements was selected in such a way that their number does not significantly slow down the calculations and, on the other hand, allows for precise mapping of body geometry and obtaining accurate results. Additionally, in order to limit the number of elements in the models, the mesh density was refined around the zones of the model which are subject to significant deformation (projectile impact points). The distance between adjacent mesh nodes ranged from 0.25 mm in the projectile impact zones to 4 mm in non-deformable armor areas. Spatial discretization was performed with the use of the HyperMesh program. The components of simulations were described by means of eight-node solid elements with reduced integration and stiffness control of the Hourglass effect. Two symmetry planes were used in the numerical model, which enabled increased accuracy of the calculations by reducing the size of the elements while preserving their number. The use of bisymmetry excluded the possibility of considering the angular velocity of the projectile. However, the data from the literature show that this velocity does not affect the obtained values of the depth of penetration of the armor, but only the degree of dispersion of the projectile fragments in case of fragmentation, which confirmed the validity of the actions taken.

**Figure 6:**
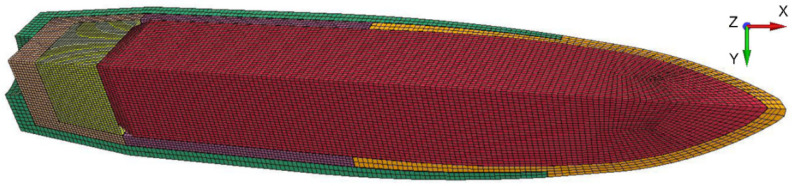
Discretization of the 7.62 mm API(BZ) projectile components.

The method of discretization of the projectile model is presented in Figure 6. It should be noted that the number of elements used was equal: 70,952 for the core, 5504 for the tombac jacket, 8576 for the steel jacket, 18,791 for the incendiary material, 8576 for the cup and 9472 for the filler. The average distance between the element nodes was ∆x = ∆y = ∆z ≈ 0.25 mm. The total number of elements in the projectile model was 121,871, which ensured both satisfactory speed and accuracy of the calculation of the single variant.

**Figure 7:**
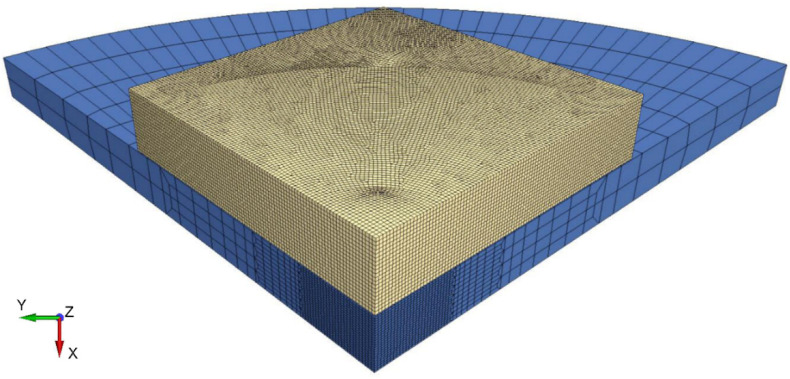
Discretization of the armor model.

The discretization of the layered armor model is shown in Figure 7. Thanks to the use of two symmetry planes, only a quarter of the armor was modeled, which limited the number of elements and accelerated the calculations.

The ceramic tile was modeled using 203,240 elements (or SPH particles in variant (3)). In addition, in variant (4) of the calculation, an algorithm of conversion of finite elements to SPH particles after exceeding defined erosion criteria was used (*DEFINE_ADAPTIVE_SOLID_TO_SPH).

The steel backing plate was modelled using 19,608 elements. The distance between the nodes varied from ∆x = ∆y = ∆z = 0.2 mm at the point of impact (20 solid elements per layer thickness) to ∆x = ∆y = ∆z = 2 mm in the layer zones distant from the point of impact (2 solid elements per layer thickness). The total number of elements in the armor model was 222,848.

The initial and boundary conditions were set in such a way that the numerical model reflected the features of the system during experimental research. The projectile was assigned an initial velocity of v_0_ = 715 m/s. The elements lying in the plane of symmetry were deprived of the possibility of displacement in the normal direction to the plane of symmetry and rotation with respect to axes lying in the plane of symmetry. The backing plate was fixed on the perimeter. The adhesive bond between the ceramic tile and the backing plate was modelled in a simplified manner by defining a tiebreak contact that broke when the stress limits (normal and shear strain =10 MPa) were exceeded. Contacts between different parts of the same body (self-contacts) were modelled using the *CONTACT-ERODING_SINGLE_SURFACE algorithm. The contact model *CONTACT-ERODING_SURFACE_TO_SURFACE was used to map mutual interactions between several bodies. The interaction between ceramic SPH particles and the finite elements of other bodies was described by the contact model *CONTACT-ERODING_NODES_TO_SURFACE. All the listed contact algorithms are based on the “penalty function” method. In this method, the contacting bodies are divided into master and slave. The distance between the nodes of the slave body and the surface of the master body in the normal direction to the segment of the master body is checked. If penetration is detected, a force is generated between the slave node and its point of contact with the master body surface to counteract this penetration. This force is dependent on the value of the penetration and the properties of the contacting bodies.

## 3. Results and Discussion

### 3.1. Analysis of the Ballistic Test Results

Ballistic tests of ceramic tiles placed on the ARMOX 500T steel backing plate were carried out in such a way that deformation of the layers caused by the previous projectiles do not coincide with deformations caused by the next projectile. After the tests at the points of impact of subsequent projectiles, the dimensions of deformation (bulge height) of the Armox 500T plate were measured. The results of ballistic tests are presented in Table 5.

In each of the five shots carried out, the projectile was stopped by the backing plate. The observed bulging of the witness plate did not exceed 3 mm. The values of the backing plate deformations were then used to determine the degree of convergence of the numerical and experimental results. The reference (average) value of the backing plate deformation (bulge height) was d = 2.5 mm.

**Figure 8:**
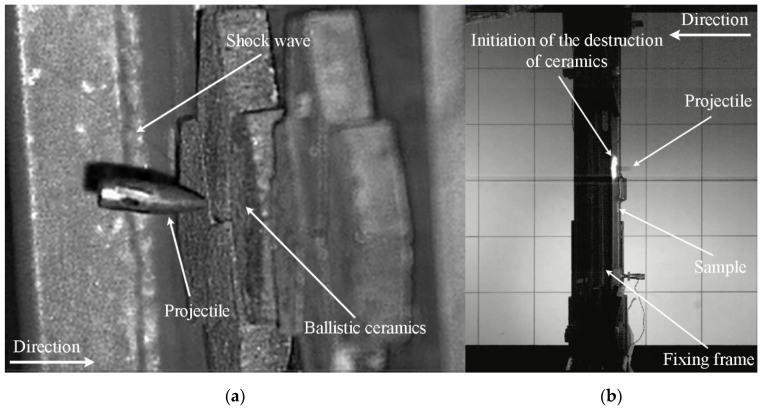
The course of the process of the projectile impact into the ceramic/steel composite armor: (**a**) the moment when the projectile is captured; (**b**) the projectile’s contact with ceramics.

The course of the projectile impact on the ceramic/steel composite armor is shown in Figure 8.

**Figure 9:**
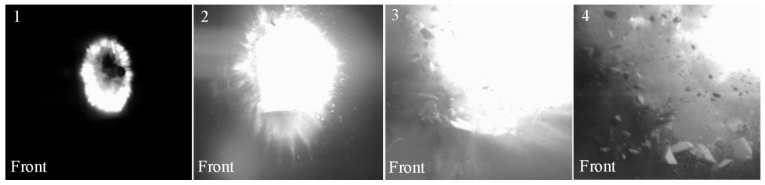
Ballistic impact test of ceramic/steel composite armor registered with a high-speed camera.

As shown in Figure 8a, the 7.62 × 39 mm API(BZ) projectile was captured at the moment of contact with the ceramic layer. During this shot, laser illumination was used and the camera was set at a certain angle, giving the wrong impression that the projectile did not maintain a 90° angle to the vertical axis of the specimen clamps. A shock wave front (characteristic Mach cone) was recorded. In Figure 8b, the image is shown as recorded perpendicular to the firing line of the sample. On the basis of the shadow photo, only the glow of the API(BZ) projectile can be seen. Around the glow of the projectile, a characteristic ring formed by the incendiary masses initiated is visible. Its flare in later stages of the penetration process makes it difficult to register the course of the phenomenon using a high-speed camera (see Figure 9). Figure 9 shows how, in step 1, a characteristic flash caused by the incendiary mass initiation could be seen. In step 2 the flash is intensified and then, in steps 3 and 4, only the poorly visible dispersion of fragments of destroyed ceramic can be noticed. However, in the final stage of the phenomenon, the degree of fragmentation of the ceramic tile can be seen—it crumbled into many small fragments. The quality of the shot recording is much worse in this case, because halogen backlighting was used. The photographic recording of the intensity of the ceramic tile fragmentation enabled subsequent quantitative verification of the simulation results.

Generally, the 5 mm-thick ceramic tile (with an areal density of about 19.9 kg/m^2^) combined with a 4 mm-thick Armox 500T steel backing plate (with an areal density of about 31.2 kg/m^2^) in each case stopped the BZ projectile without perforation. It should be noted that, in the case of monolithic steel structures, the same effect is obtained (the 7.62 × 39 mm API(BZ) projectile is effectively stopped) by a 12mm-thick steel armor plate with a hardness of about 500 HBW and areal density of about 94.2 kg/m^2^ [49].

### 3.2. Analysis of Numerical Results

**Figure 10:**
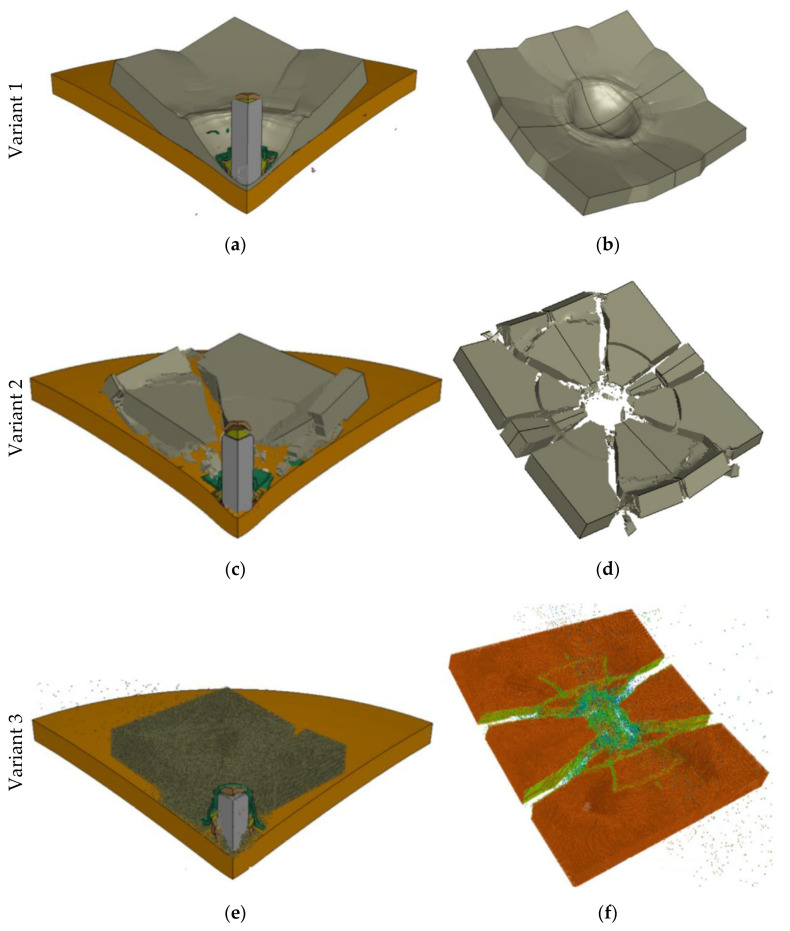

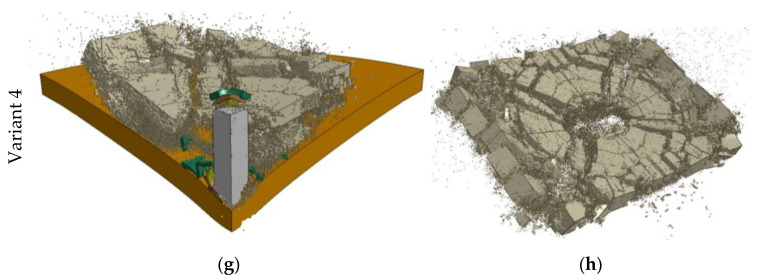
Deformations of the simulation components and the 5mm-thick Al_2_O_3_ ceramic tile for time t = 0.125 ms: (**a**,**b**) Finite element method without erosion; (**c**,**d**) Finite element method with erosion; (**e**,**f**) SPH method; (**g**,**h**) Hybrid elements (conversion of FEM to SPH after destruction).

The results of numerical analysis of the ceramic/steel composite armor impacted by the 7.62 × 39 mm API(BZ) projectile were presented in the form of deformations of the components (see Figure 10). The numerical simulations enabled a more accurate analysis of the phenomena occurring during the process of armor penetration than in the case of experimental research. In spite of different modelling methods used in particular calculation variants, the general course of the penetration process is similar. In the first stage the projectile hit the armor and a compressive shock wave was generated, which moved in the direction of impact. The high-yield point and hard surface of the ceramic tile caused crushing of the tip of the projectile core and partial scattering of the moment transmitted by the projectile. When the wave reached the rear surface of the ceramic tiles, it was reflected as a tensile wave. As a result, cracks were generated in the ceramic tile in the shape of a characteristic cone. The velocity of the front of the projectile was much lower than that of the rear side. This difference in velocity caused an increase in compressive stress in the projectile and its erosion.

In the second stage, the projectile penetrated the armor at a relatively constant velocity. The ceramic tile fragments were pushed out radially and the elastic deformation of the armor base began. The tip of the projectile eroded, which resulted in a further increase in stress along the length of the projectile. In the last stage of the penetration process the projectile penetrated the steel plate. The residual energy of the projectile and the fragments of ceramic plate was absorbed by the steel layer. The penetrating projectile changed the form of plastic deformation of the armor material from shearing to bulging of the armor steel plate surface. At this stage the projectile was further damaged and its velocity was decreased. In the final phase of the penetration process, tensile stress appeared in the projectile instead of the previously occurring compressive stress, which caused further fragmentation of the projectile core.

Numerical simulations have been performed to compare the effectiveness of the available techniques of modeling ceramic tiles as well as to determine the extent to which individual numerical models reproduce the actual behavior of materials and components observed during experimental studies. First of all, the presence or absence of perforation was assessed. Among the other evaluated parameters, some were of qualitative nature, such as the assessment of the way ceramic tiles cracked during ballistic impact or the intensity of erosion of the projectile core. The degree of correlation between the numerical and experimental results was assessed for such parameters in a subjective manner. The scale for qualitative parameters included the following evaluations of the degree of correlation:

1. Very good correlation;
2. Satisfactory correlation;
3. Poor correlation;
4. No correlation.

When comparing the numerical values (quantitative evaluation, e.g., the bulge height of the backing plate) the relative error value was the measure of convergence between the simulation and experimental results. Similarly to qualitative parameters, the following scale of correlation between simulation and experimental results was adopted (on the basis of previous experience of the authors’ team in the field of numerical simulation of ballistic impact on ceramic tiles) [42,50]:

1. Very good correlation for relative error ∆e ≤ 10%;
2. Satisfactory correlation for relative error ∆e = 11÷25%;
3. Poor correlation for relative error ∆e = 26÷40%;
4. No correlation for relative error ∆e > 41%.

In all of the analyzed cases, armor with Al_2_O_3_ ceramic frontal layers stopped the projectile in a similar way as during experimental research.

Figure 10 shows a comparison of the obtained results of numerical analyses in terms of qualitative representation of the degree of fragmentation of ceramic tiles.

The greatest similarity to the experimental character of ceramic cracking was observed for variant 4 (Figure 10h), in which hybrid elements were used. As in the experiment (see Figure 9), the layer crumbled into many small fragments during the projectile impact, which indicates a very good correlation. On the contrary, a lack of correlation can be seen in variant 1 (see Figure 10b). A satisfactory correlation was obtained in variants 2 and 3 (see Figure 10d,f).

**Figure 11:**
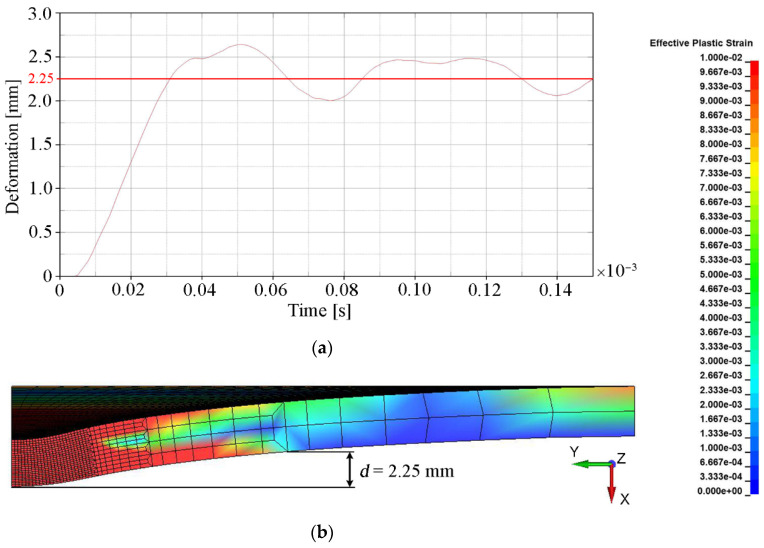
Deformation values of the backing plate for variant 1: (**a**) Course of deformation values for time t = 1.5 × 10^−4^ s; (**b**) Distribution of plastic strain.

**Figure 12:**
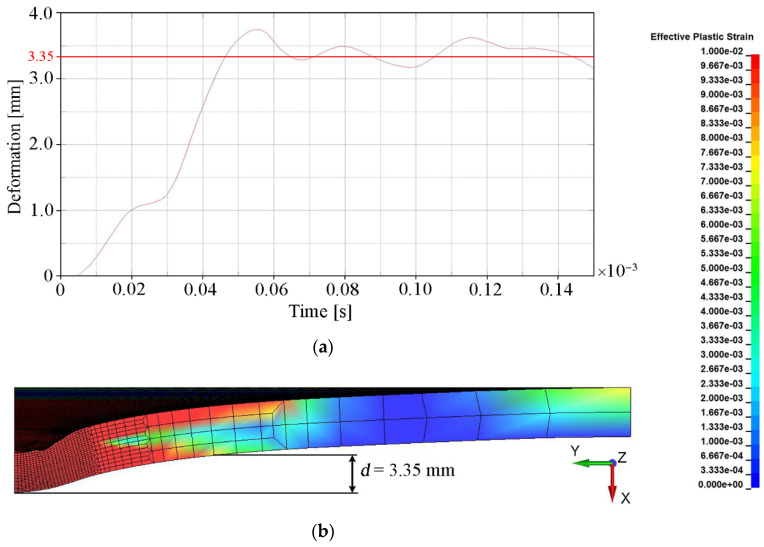
Deformation values of the backing plate for variant 2: (**a**) Course of deformation values for time t = 1.5 × 10^−4^ s; (**b**) Distribution of plastic strain.

**Figure 13:**
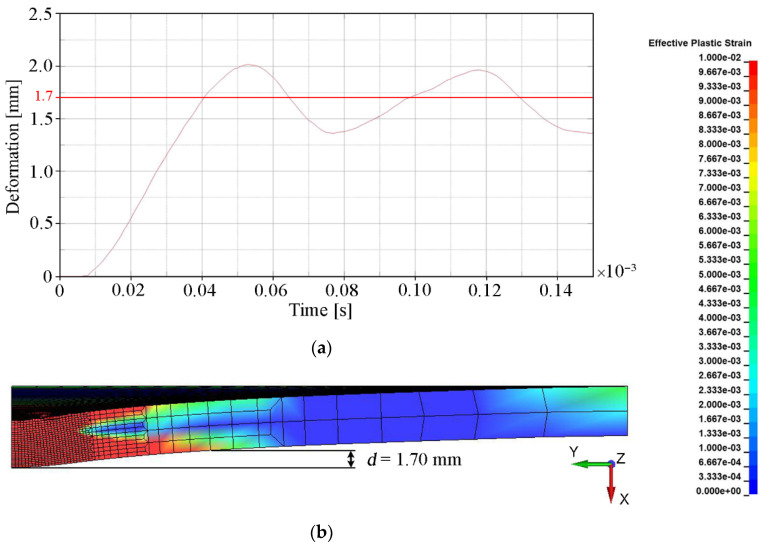
Deformation values of the backing plate for variant 3: (**a**) Course of deformation values for time t = 1.5 × 10^−4^ s; (**b**) Distribution of plastic strain.

**Figure 14:**
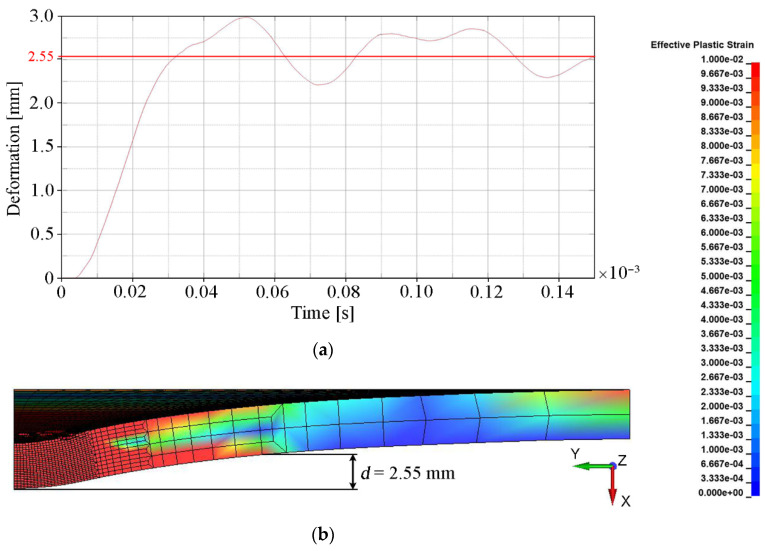
Deformation values of the backing plate for variant 4: (**a**) Course of deformation values for time t = 1.5 × 10^−4^ s; (**b**) Distribution of plastic strain.

The main quantitative parameter determining the degree of convergence of the numerical solution with the experimental results (see Table 5) was deformation, understood as the bulge height of the backing plate. This parameter indirectly determines how much of the projectile energy was absorbed by the ceramic tile during the penetration process. The deformation of the backing plate was determined when the calculation was interrupted, i.e., t = 0.15 × 10^−3^ s. The backing plate did not reach a state of equilibrium and had a certain elasticity. Therefore, in order to estimate the final value of its deformation, the area where the plate was subject to any plastic deformation was determined (ε_p_ = 0.01 was chosen as a minimum value). The measure of the deformation was the distance (measured in the normal direction to the initial surface of the plate) between the central point of the rear surface of the steel armor plate and the point on the border of the plastic deformation area (Figure 11b). The value around which the course of the backing plate deformation dependence oscillated in time was the bulge height of the backing plate. Figure 11, Figure 12, Figure 13 and Figure 14 show the bulge heights of the backing plate achieved in numerical analyses.

The smallest deformations of the steel plate were observed for variant 1-FEM without erosion (see Figure 11) with a value of 2.25 mm and variant 3 SPH method (see Figure 13) with a value of 1.70 mm. The protective effectiveness of the ceramic tile is in both cases overestimated, in variant 1 as a result of lack of representation of the cracking mechanism of Al_2_O_3_ (see Figure 10b) and in variant 3 as a result of interaction between particles, which prevents their stretching (see Figure 10f). The highest deformations were observed in variant 2 (see Figure 12), where the bulge height was 3.35 mm.

As a result of finite element erosion, a significant mass of the ceramic tile is removed. This does not allow effective reproduction of the mechanisms of projectile resistance in the ceramic tiles and, consequently, the effectiveness of the ceramics in projectile resistance was thus underestimated. The results closest to the real ones were estimated for variant 4 (see Figure 14), where a deformation value of 2.55 mm was obtained.

This value is closest to the reference experimental result (see Table 5), i.e., 2.50 mm (relative error of Δ = 2%).

Thanks to the application of the hybrid method (finite elements converted into SPH particles after exceeding defined failure criteria), the following key physical phenomena occurring during the penetration of armor containing ceramic tiles (see Figure 10g,h) with AP projectiles were reproduced:

- Formation of radial and circumferential cracks in ceramics;
- Formation of Hertzian cones in ceramics;
- Crushing of the tip of the AP projectile core by the ceramic tile, thus reducing its penetration effectiveness.

The greatest differences from the experiments (relative error Δ = 34%) were obtained in variant 2 of simulation (FEM with erosion). This was due to erosion of a large number of degenerated finite elements. As a consequence, the protective capacity of the ceramic tile was underestimated and the amount of energy absorbed by the plastic deformation of the backing plate increased.

**Figure 15:**
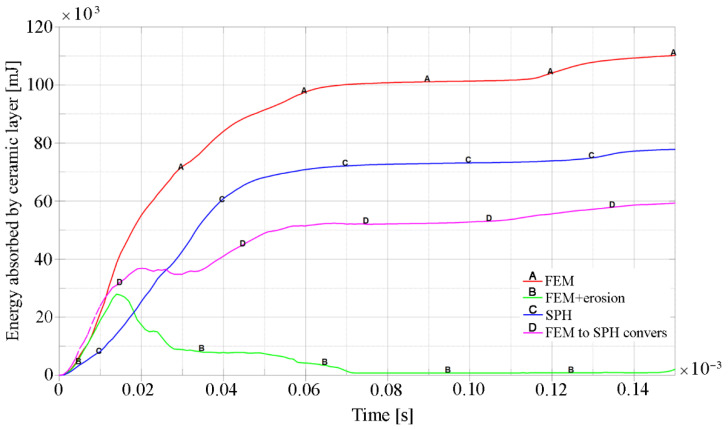
Change in the amount of absorbed energy of the ceramic tile over time for individual calculation variants.

The conclusions drawn from the deformations of the backing plate were confirmed also by the graph showing the energy absorbed by the ceramic tile as a function of time for different techniques of ceramic tile modeling (see Figure 15).

The highest amount of energy was absorbed by the ceramic tile modeled with FEM without erosion. Since there were no erosion criteria defined, the cracks in the ceramic tile were not generated, and there was no fragmentation of the layer, which caused overestimation of the protective capabilities of the ceramics. On the contrary, in the variant of FEM with erosion, the final amount of absorbed energy was close to zero. It was caused by deletion of the elements that absorbed part of the energy of the projectile, and in this way, were subjected to deformations exceeding the defined erosion criteria. The beginning of the erosion process is clearly visible on the graph after the time t = 0.013 ms. In the variant where a hybrid method of modeling of the ceramic tile was used, a moment when the erosion process started is also visible (t = 0.014 ms). However, in this case, the failed elements were replaced by the SPH particles. Thanks to this, the ceramic layer could absorb additional amounts of energy by generation and propagation of cracks as well as by the interaction of the projectiles with damaged ceramic fragments. The variants where SPH and hybrid elements were used exhibit the greatest correlation with the experimental observations. However, the process of fragmentation of the ceramic tile is much more similar to that observed in the experiments in the variant where hybrid elements were used (see Figure 10h) than in the variant where SPH particles were used (see Figure 10f).

**Figure 16:**
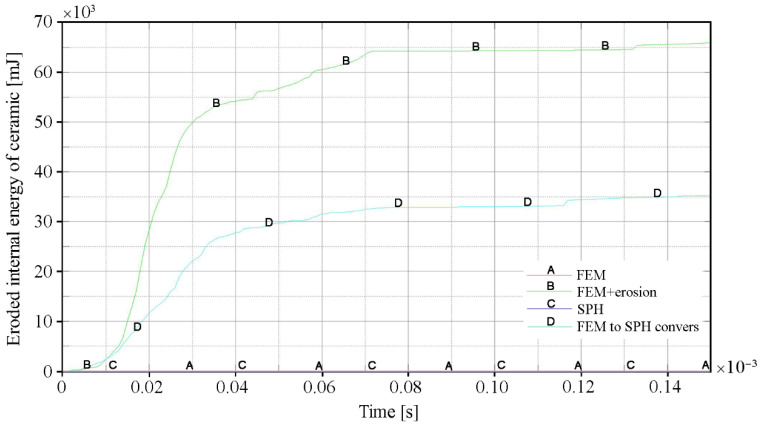
Change in the amount of eroded internal energy of the ceramic tile over time for individual calculation variants.

In Figure 16, the change in the amount of eroded internal energy inside the ceramic tile over time is shown. For variants 1 (FEM) and 3 (SPH), the amount of this energy was equal to zero because erosion algorithms were not used. In variant 4 (FEM to SPH conversion) the number of elements is over two times lower than in variant 2 (FEM with erosion). In addition, in variant 4, the eroded elements were replaced by SPH particles, so the protective effectiveness of the ceramic tile was not artificially underestimated by erosion.

**Figure 17:**
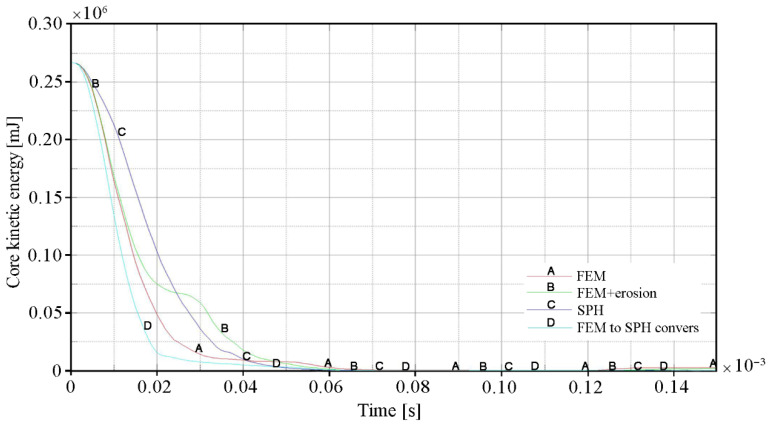
Change in kinetic energy of the projectile core over time for individual calculation variants.

**Figure 18:**
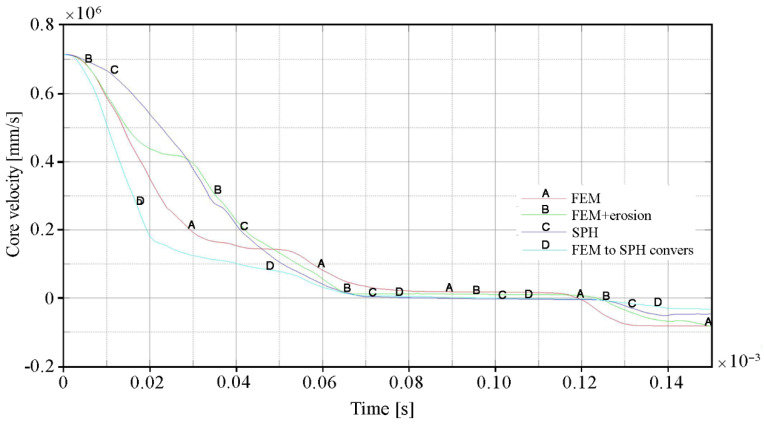
The time course of the projectile core velocity values for individual calculation variants.

The influence of the erosion algorithm on the penetration process of ceramic/steel composite armor is also clearly visible in Figure 17 and Figure 18, where the changes in kinetic energy and velocity of the projectile core in time are shown respectively. The curves for variant 2 of the calculation (FEM with erosion) have a characteristic stepped shape. This was a result of the increase in stress and, as a consequence, premature excessive erosion of a certain volume of the ceramic tile located in the area of direct impact in the projectile tip. Therefore, during the period t = 0.02 ÷ 0.04 × 10^−3^ s, the projectile was not decelerated and moved at a relatively constant velocity. Further deceleration occurred only after interaction with the backing plate had started. The curve patterns of the other calculation variants were much more similar to the real one. Figure 17 and Figure 18 clearly show that the most intense deceleration of the projectile occurred in variant 4 (FEM to SPH conversion). The curve for Variant 3, in which the ceramic tile was modelled with SPH particles, has the smoothest shape. However, in this case, this was not due to the lowest protective effectiveness of the ceramic tile, but only to the most intense destruction and erosion of the projectile core.

The results of numerical analyses for individual techniques of modeling of ceramics under ballistic impact conditions and the degree of correlation between simulation and experimental results were presented in Table 6.

Summarizing the data in Table 6, it can be concluded that the simulation results closest to the experimental observations can be obtained by using a hybrid technique of modeling ceramic tiles using the conversion of finite elements to SPH particles after reaching the defined failure criteria. It provides the best representation of the process of cracking of ceramics under ballistic impact conditions and, as a result, allows us to determine the protective effectiveness of armor containing ceramic tiles with the highest accuracy among the analyzed methods.

**Figure 19:**
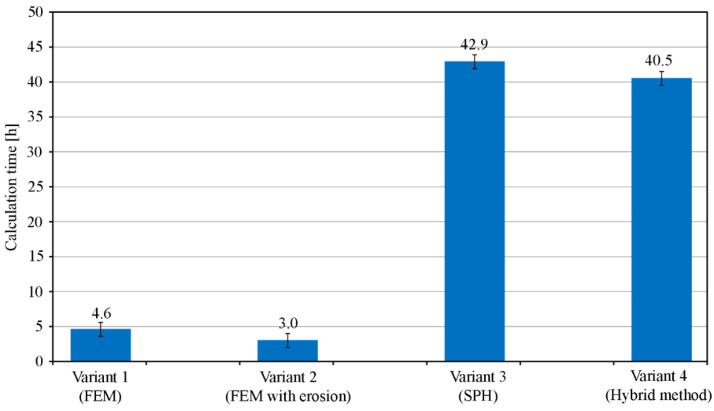
Comparison of calculation time for individual variants.

Figure 19 shows a comparison of the time necessary to complete the calculations for each variant.

The calculations were carried out on 7 cores with 3.2 GHz clock and 10 Gb of operating memory. It is clear that introducing SPH particles into the model significantly slows down the calculations (calculation time about 10× longer). Taking into account the quality of the reproduction of the phenomenon, the hybrid method (variant 4 —conversion of finite elements to SPH particles) gives the best results and should be used in cases where accurate results are required. In other situations, where the purpose of calculations is not to determine, for example, the exact extent of penetration depth, but only to observe a certain trend, the FEM method with defined erosion criteria (variant 2), which is more than 10× faster, can be considered for use.

## 4. Conclusions

The effectiveness of available numerical techniques in mapping the characteristic behavior of ballistic ceramics under projectile impact conditions was analyzed and compared in this article. Particular attention was paid to reproduction of the phenomena of generation and propagation of cracks in ceramics, which is crucial for definition of an accurate numerical model of the ballistic impact phenomenon on ceramic layers. On the basis of the results of experimental tests and numerical simulations, the following conclusions can be drawn:

1. The Al_2_O_3_ ceramic tile with a thickness of 5 mm and an areal density of about 19.4 kg/m^2^, together with the Armox 500T steel backing plate with a thickness of 4 mm and an areal density of 31.2 kg/m^2^, protect against the 7.62 × 39 mm API(BZ) projectile;
2. Numerical models defined in the work provide a satisfactory degree of representation of the actual behavior of both the 7.62 × 39 API(BZ) projectile and the composite ceramic-metal armor under ballistic impact conditions;
3. A ceramic tile simulation modeled with hybrid elements (i.e., finite elements converted into SPH particles) outperforms other simulation techniques, and can be used for preliminary estimation of the protective effectiveness of the analyzed layered ceramic/steel armor using computer simulation methods;
4. The FEM method with defined erosion criteria is more than 10 times faster than the hybrid simulation techniques, but the results could be highly imprecise. Therefore, this method can be used only in cases where the purpose of the calculation is not to determine, for example, the exact extent of penetration depth, but only to observe a certain trend;
5. The results of numerical simulations with the FEM method without erosion showed the lowest correlation with the experimental observations, and should not be used.

## Figures and Tables

**Table 1:** Properties of special ceramics in comparison with RHA [35].

Specification	Density, *ρ* [g/cm^3^]	Young’s Molus, E [GPa]	Tensile Strength, R_m_ [MPa]	Knopp Harness, H_K_ [GPa]	Melting Point, T_t_ [K]	Ballistic Resistance, [K/kg × 10^3^]
Al_2_O_3_	3.9	390	370	18	2320	1.5
SiC	3.1	410	200	21	3300	1.8
B_4_C	2.5	450	300	30	3300	5.3
TiB_4_	4.5	570	350	33	3230	5.0
RHA	7.8	210	1000	3.5	1950	0.5

**Table 2:** Mechanical properties and chemical composition of # 4 mm ARMOX 500T armor steel [36,37].

Specification		Yield Strength, R_p0.2_ [MPa]		Tensile Strength, R_m_ [MPa]		Hardness, HBW			Elongation, A_5_ [%]		Charpy-V, KCV_−40 °C_ [J/cm^2^]		Charpy-V, KCV_+20 °C_ [J/cm^2^]	
A500T *		1442		1625		512			13		104		185	
A500T		1240		1450–1750		480–540			min. 8		min. 32		-	
**Chemical Composition [%]**														
C	Mn		Si		P		S	Cr		Ni		Mo		B
0.280 *	0.860 *		0.260 *		0.006 *		0.001 *	0.500 *		0.870 *		0.352 *		0.002 *
0.320	1.200		0.400		0.010		0.003	1.000		1.800		0.700		0.005

* Own research results.

**Table 3:** Parameter values of the J–C models.

Parameter	Hardened Core	Tombac Jacket	Steel Jacket	Lead Jacket	Incendiary Mass	Armox 500T Plate
*ρ* [g/cm^3^]	7.85	8.96	7.85	11.34	2.00	7.85
E [GPa]	210	124	210	16	6	210
v [-]	0.33	0.34	0.33	0.42	0.30	0.33
C_p_ [J/kgK]	-	3850	4770	-	-	4770
T_m_ [K]	-	1356	1800	-	-	1800
Strength model	*MAT_098	*MAT_107	*MAT_107	*MAT_098	*MAT_098	*MAT_107
A [MPa]	1976	206	448	24	78	1580
B [MPa]	2856	505	303	300	160	756
n [-]	0.207	0.310	0.150	1.000	1.000	0.199
C [-]	0.005	0.025	0.003	0.100	0.0	0.005
m [-]	-	1.09	1.03	-	-	0.81
**Failure Model J–C**						
D_1_	-	0.540	0.540	-	-	0.068
D_2_	-	4.88	4.88	-	-	5.32
D_3_	-	−3.03	−3.03	-	-	−2.55
D_4_	-	0.014	0.014	-	-	0.016
D_5_	-	1.12	1.12	-	-	1.10
*MAT_ADD EROSION	MNPRES = −2600 EPSSH = 1 VOLEPS = 0.05	VOLEPS = 0.2 EPSSH = 1	VOLEPS = 0.2 EPSSH = 1	VOLEPS = 0.5 EPSSH = 1	VOLEPS = 0.01 EPSSH = 1	EPSSH = 1

**Table 4:** Values of parameters in the *MAT_110-Johnson_Holmquist_ Ceramic model.

Parameter	Al_2_O_3_
*ρ* [g/cm^3^]	3.84
G [GPa]	93
A [-]	0.93
B [-]	0.31
C [-]	0.007
m [-]	0.6
n [-]	0.64
EPSI	1
T [MPa]	262
SFMAX	1
HEL [MPa]	8000
PHEL [MPa]	1460
Beta	1
D_1_ [-]	0.01
D_2_ [-]	0.7
K_1_ [GPa]	131
K_2_ [GPa]	0
K_3_ [GPa]	0
*MAT_ADD_EROSION	VOLEPS 0.05

**Table 5:** Results of ballistic impact of the 7.62 × 39 mm API(BZ) projectile on Al_2_O_3_ ceramic tiles placed on Armox 500T.

Shots No.	Projectile Velocity [m/s]	Penetrations/ None	Deformations [mm]	Remarks
1	703.6	None	2.5	
2	699.4	None	2.0	
3	708.3	None	2.5	
4	712.1	None	3.0	Little crack on the rear side of backing plate
5	703.4	None	2.5	

**Table 6:** Summary of obtained results of numerical analyses and their degree of correlation with experimental results.

Parameter	Experiment	Variant 1 (FEM)	Variant 2 (FEM + Erosion)	Variant 3 (SPH)	Variant 4 (FEM to SPH Conversion)
Ceramic tile fragmentation intensity	High intensity (large number of small fragments)	No fragmentation No correlation	Low Intensity Acceptable correlation	Low Intensity Acceptable correlation	High Intensity Very strong correlation
Backing plate deformation [mm]	2.5 mm	2.25 mm Error Δ = 11% Acceptable correlation	3.35 mm Error Δ = 34% Weak correlation	1.70 mm Error Δ = 32% Weak correlation	2.55 mm Error Δ = 2% Very strong correlation

## Data Availability

Data sharing is not applicable to this article.

## References

[1] Hazell P.J. (2016). Armor: Materials, Theory and Design.

[2] Mahesh V., Mahesh V., Harursampath D. (2021). Ballistic characterization of fiber elastomer metal laminate composites and effect of positioning of fiber reinforced elastomer. Proc. Inst. Mech. Eng. Part L J. Mater..

[3] Mahesh V., Joladarashi S., Kulkarni S.M. (2021). Comparative study on ballistic impact response of neat fabric, compliant, hybrid compliant and stiff composite. Thin Walled Struct..

[4] Mahesh V., Joladarashi S., Kulkarni S.M. (2019). An experimental investigation on low-velocity impact response of novel jute/ rubber flexible bio-composite. Compos. Struct..

[5] Bhatnagar A. (2016). Lightweight Ballistic Composites. Military and Law-Enforcement Applications.

[6] Reddy P.R.S., Savio S.G., Madhu V., Mahajan Y., Johnson R. (2019). Ceramic composite armour for ballistic protection. Handbook of Advanced Ceramics and Composites.

[7] Medvedovski E. (2010). Ballistic performance of armour ceramics: Influence of design and structure. Part 1. Ceram. Int..

[8] Healey A., Cotton J., Maclachlan S., Smith P., Yeomans J. (2017). Understanding the ballistic event: Methodology and initial observations. J. Mater. Sci..

[9] Jones T.L., Vargas-Gonzalez L.R., Scott B., Goodman B., Becker B. (2020). Ballistic evaluation and damage characterization of 3-D printed, alumina-based ceramics for light armor applications. Int. J. Appl. Ceram. Technol..

[10] Akella K., Naik N.K. (2015). Composite armour—A review. J. Indian Inst. Sci..

[11] Crouch G. (2017). The Science of Armor Materials.

[12] Braga F.O., da Luz F.S., Monteiro S.N., Lima E.P. (2018). Effect of the impact geometry in the ballistic trauma absorption of a ceramic multilayered armor system. J. Mater. Res. Technol..

[13] Gositanon A., Chaiyarit M., Phabjanda S. (2018). Ballistic simulation and verification of ceramic/rubber composite armor. 6th International Conference on Mechanical, Automotive and Materials Engineering (CMAME).

[14] Luo D., Wang Y., Wang F., Cheng H., Zhu Y. (2019). Ballistic behavior of oblique ceramic composite structure against long-rod tungsten projectiles. Materials.

[15] Stanislawek S., Morka A., Niezgoda T. (2015). Pyramidal ceramic armor ability to defeat projectile threatby changing its trajectory. Bull. Pol. Acad. Sci. Tech. Sci..

[16] Gonzalez-Albuixech V.F., Rodrıguez-Millan M., Ito T., Loya J.A., Miguelez M.H. (2019). Numerical analysis for design of bioinspired ceramic modular armors for ballistic protections. Int. J. Damage Mech..

[17] Miranda P., Pajares A., Meyers M.A. (2019). Bioinspired composite segmented armor: Numerical simulations. J. Mater. Res. Technol..

[18] Ben-Dor G., Dubinsky A., Elperin T. (2018). Optimization of ballistic properties of layered ceramic armor with a ductile back plate. Mech. Based Des. Struct. Mach..

[19] Savio S.G., Rao A.S., Rama Subba Reddy P., Madhu V. (2019). Microstructure and ballistic performance of hot pressed & reaction bonded boron carbides against an armor piercing projectile. Adv. Appl. Ceram..

[20] Rosenberg Z., Dekel E. (2020). Terminal Ballistics.

[21] Yang L., Chen Z., Dong Y., Zi F., Yang J., Wu L. (2020). Ballistic performance of composite armor with dual layer piecewise ceramic tiles under sequential impact of two projectiles. Mech. Adv. Mater. Struct..

[22] Baranowski P., Kucewicz M., Gieleta R., Stankiewicz M., Konarzewski M., Bogusz P., Pytlik M., Malachowski J. (2020). Fracture and fragmentation of dolomite rock using the JH-2 constitutive model: Parameter determination, experiments and simulations. Int. J. Impact Eng..

[23] Donncha L., William R., O’DonoghuePadraic E., Leen S.B. (2019). A review of the integrity of metallic vehicle armor to projectile attack. Proc. I. Mech. E. Part. L J. Mater. Des. Appl..

[24] Neckel L., Hotza D., Stainer D., Janssen R., Al-Quresh H.A. (2013). Simulation and optimization in materials technology. Adv. Mater. Sci. Eng..

[25] Forental G.A., Sapozhnikov S.B. (2016). Effective FEA design of hard face composite structures to stop armor piercing projectiles. WIT Trans. Built Environ..

[26] Chabera P., Boczkowska A., Morka A., Niezgoda T., Ozieblo A., Witek A. (2014). Numerical and experimental study of armor system consisted of ceramic and ceramic- elastomer composites. Bull. Pol. Acad. Sci. Tech. Sci..

[27] Rahbek D.B., Simons J.W., Johnsen B.B., Kobayashi T., Shockey D.A. (2017). Effect of composite covering on ballistic fracture damage development in ceramic plates. Int. J. Impact Eng..

[28] Kurzawa A., Pyka D., Jamroziak K., Bajkowski M., Bocian M., Magier M., Koch J. (2020). Assessment of the impact resistance of a composite material with EN AW-7075 matrix reinforced with α-Al_2_O_3_ particles using a 7.62 × 39 mm projectile. Materials.

[29] Clayton J.D. (2015). Modeling and simulation of ballistic penetration of ceramic-polymer-metal layered systems. Math. Probl. Eng..

[30] Fras T., Roth C.C., Mohr D. (2020). Application of two fracture models in impact simulations. Bull. Pol. Acad. Sci. Tech. Sci..

[31] Islam M.R.I., Peng C. (2019). A total Lagrangian SPH method for modelling damage and failure in solids. Int. J. Mech. Sci..

[32] Xiao Y., Wu H., Ping X. (2020). On the simulation of fragmentation during the process of ceramic tile impacted by blunt projectile with SPH method inLS-DYNA. CMES-Comp. Model. Eng..

[33] Becker M., Seidl M., Mehl M., Souli M. Numerical and experimental investigation of SPH, SPG, and FEM for high-velocity impact applications. Proceedings of the 12th European LS-DYNA Conference 2019.

[34] Heimbs S. (2011). Computational methods for bird strike simulations: A review. Comput. Struct..

[35] Cegła M. (2018). Special ceramics in multilayer ballistic protection systems. Issues Armament Technol..

[36] Jamroziak K., Konat L., Bocian M., Pekalski G. Structural aspects of the ballistic impact test illustrated by steel-cored missile and by rolled homogeneous steel armor. Proceedings of the 6th Interantional Armament Conference, SAAT 2006.

[37] (2017). Operation Sheet, Armox 500T, ARMOX® Protection Plate, SAAB. https://www.ssab.pl/api/sitecore/Datasheet/GetDocument?productId=ACD3681501884BA2B09D742FE19A0F7F&language=pl-PL.

[38] AEP-55, Volume 1 (Edition 1). Procedures for Evaluating the Protection Level of Logistic and Light Armored Vehicles. http://www.englands1.com/site/wp-content/uploads/AEP-55.pdf.

[39] Johnson G.R., Cook W.H. (1985). Fracture characteristics of three metals subjected to various strains, strain rates, temperatures and pressures. Eng. Fract. Mech..

[40] Poplawski A., Kedzierski P., Morka A. (2020). Identification of Armox 500T steel failure properties in the modeling of perforation problems. Mater. Design.

[41] Burian W., Zochowski P., Gmitrzuk M., Marcisz J., Starczewski L., Juszczyk B., Magier M. (2020). Protection effectiveness of perforated plates made of high strength steel. Int. J. Impact Eng..

[42] Zochowski P. (2017). Numerical-Experimental Evaluation of Protection Capability of Armors Containing Layers Made of Nanostructured Steels.

[43] Serjouei A., Chi R., Sridhar I., Tan G.E.B. (2015). Empirical ballistic limit velocity model for bi-layer ceramic–metal armor. Int. J. Prot. Struct..

[44] Iqbal M.A., Senthil K., Sharma P., Gupta N.K. (2016). An investigation of the constitutive behavior of Armox 500T steel and armor piercing incendiary projectile material. Int. J. Impact Eng..

[45] Wisniewski A., Zochowski P. (2013). Building and validation of numerical models of the B-32 type armor-piercing projectiles. Probl. Mechatroniki.

[46] Golewski P., Rusinek A., Sadowski T. (2020). Material Characterization of PMC/TBC Composite Under High Strain Rates and Elevated Temperatures. Materials.

[47] Feli S., Asgari M.R. (2011). Finite element simulation of ceramic/composite armor under ballistic impact. Compos. Part B-Eng..

[48] Bresciani L.M., Manes A., Romano T.A., Iavarone P., Giglio M. (2016). Numerical modelling to reproduce fragmentation of a tungsten heavy alloy projectile impacting a ceramic tile: Adaptive solid mesh to the SPH technique and the cohesive law. Int. J. Impact Eng..

[49] Jamroziak K. (2010). Assessment of Impact Resistance of Armor Panels According to EN PN 1522 Standard. Research Report for the Project No. 381/BO/B—Construction of MRAP Class Armored Vehicle with Increased Resistance to Mines and Explosives at the Base of Chassis Mercedes Benz Unimog.

[50] Zochowski P. (2017). Nanostructured Steels in Composite Armors. PhD Thesis.

